# Echocardiographic evaluation of non-surgically treated mild-to-moderate mitral dysfunction in patients undergoing aortic valve replacement

**DOI:** 10.1186/s13019-019-0934-7

**Published:** 2019-06-20

**Authors:** Gwan Sic Kim, Joon Bum Kim, Suk Jung Choo, Cheol Hyun Chung, Jae Won Lee, Sung-Ho Jung

**Affiliations:** 1 Department of Thoracic and Cardiovascular Surgery, Ulsan University Hospital, University of Ulsan College of Medicine, 877, Bangeojinsunhwando-ro, Dong-gu, Ulsan, 44033 Republic of Korea; 2 Department of Thoracic and Cardiovascular Surgery, Asan Medical Center, University of Ulsan College of Medicine, 88, Olympic-ro 43-gil, Songpa-gu, Seoul, 138-736 Republic of Korea

**Keywords:** Aortic valve, Mitral valve, Replacement

## Abstract

**Background:**

Surgical management of the mitral valve (MV) in patients with mild-to-moderate mitral dysfunction undergoing aortic valve replacement is still controversial. We investigated the echocardiographic data from patients with mild-to-moderate mitral dysfunction who did not undergo MV surgery.

**Materials and methods:**

From January 1989 to June 2012, a total of 2731 patients underwent aortic valve replacement. Among these, 560 patients with mild-to-moderate mitral dysfunction were screened. Of these, 292 patients (61.9 ± 13.0 years; 113 females) who had not undergone MV surgery formed our study cohort. Survival, valve-related complication, and echocardiographic data were evaluated.

**Results:**

There were three early deaths. During the mean follow-up period of 56.9 ± 46.5 months, there were 23 late deaths and 28 valve-related complications. Valve-related event-free survival at 5 years was 85.9% ± 2.4%. In serial postoperative echocardiographic evaluations (mean follow-up duration: 40.8 ± 44.5 months), 21 patients experienced a progression in late mitral dysfunction. At 5 years, 88.8% ± 2.7% of patients did not suffer from late mitral dysfunction. Based on multivariate analysis, rheumatic pathology of MV (Hazard Ratio: 3.88, 95% confidence intervals 1.60–9.39, *p* = 0.003) was an independent predictor of late mitral dysfunction.

**Conclusions:**

Conservatively treated patients with mild-to-moderate mitral dysfunction exhibited acceptable clinical outcomes. Rheumatic pathology of MV is associated with a higher risk of progressive native MV dysfunction.

## Introduction

Aortic valve replacement (AVR) is the most frequently performed valve surgery. Patients undergoing AVR often present with a concomitant mitral valve (MV) pathology [1–4]. In the presence of severe mitral disease, concomitant MV surgery with AVR is generally considered. However, in less severe cases of MV pathology, the decision to perform MV surgery remains controversial.

Conservative management of less severe MV disease has many benefits, including the avoidance of unnecessary MV surgery [5–9], a shorter aortic cross/operation time, and consequently a lower perioperative morbidity and mortality than double valve surgery [10, 11]. However, this conservative approach may lead to a higher chance of reoperation resulting from unchanged or worsened mitral pathology [12, 13] and lesser hemodynamic improvement than surgery [14].

Because of limited data and the lack of randomized control trials, even the present 2014 ACC/AHA guidelines do not provide clear recommendations for managing MV disease [15]. In 2013, some studies provided relevant clinical insights into this disease [14, 16]. However, there is still insufficient evidence to resolve the issue, especially regarding organic MV pathology and the paucity of long-term echocardiographic data [6, 12, 13, 17, 18].

The natural course of non-surgically treated mild-to-moderate mitral dysfunction in patients undergoing AVR still remains to be elucidated. Therefore, we investigated the long-term echocardiographic data of patients with mild-to-moderate mitral dysfunction including organic MV pathology after isolated AVR in terms of clinical outcomes and mitral dysfunction. We also sought to determine the risk factors associated with progressive mitral dysfunction.

## Materials and methods

### Patients

From January, 1989, to June, 2012, 2731 patients underwent AVR at the Asan Medical Center in Seoul, South Korea. Of these, 752 patients with mild-to-moderate mitral dysfunction, defined as mild-to-moderate mitral regurgitation (MR) or mitral stenosis of 1.1 cm2 ≤ MV area ≤ 2.0 cm2, were screened. Among these, patients with associated valve lesions (pulmonary valve replacement, tricuspid valve replacement) or previous aortic surgery, coronary artery bypass grafting, or redo-surgery were excluded. However, patients who had undergone concomitant tricuspid repair were not excluded. Ultimately, 560 patients with mild-to-moderate mitral dysfunction were identified, of which 292 patients had not undergone MV surgery and 268 patients had undergone combined MV surgery. Whether or not to perform mitral valve surgery was determined by individual surgeon preference. In this study, we reviewed a total of 292 patients who had not undergone MV surgery. Survival, valve-related complication, and echocardiographic data were evaluated. This study was approved by our institutional review board, which waived the requirement for informed patient consent, based on the retrospective nature of the study.

### Surgical procedures

A median sternotomy approach along with conventional ascending aorta and bicaval cannulations were used for all patients. Moderately hypothermic cardiopulmonary bypasses were used, and myocardial protection was achieved with cold or tepid blood cardioplegia. After aortic cross-clamping, the aorta was opened either with a transverse or oblique aortotomy. Morphology of the aortic valve was then inspected and excision of the valve was performed. The aortic valve was replaced with a mechanical valve (*n* = 163) or a tissue valve (*n* = 129). The Maze procedure (*n* = 9) was performed using a modified Cox-Maze III procedure. Since February 2006, the Maze procedure has been performed by cryoablation using a flexible probe, SurgiFrost (Medtronic, Minneapolis, MN).

### Postoperative anticoagulation

Patients who underwent bioprosthetic valve implantation were routinely administered warfarin for 3–6 months postoperatively, with a target international normalized ratio (INR) of 2.0–2.5. The maintenance of anticoagulation therapy thereafter was determined according to the presence of thromboembolic risks and cardiac rhythm status in each patient. For patients with mechanical valve implantation, an INR of 2.0 to 2.5 was targeted.

### Follow-up

Follow-up data were obtained from hospital records, clinical visits, and telephone interviews. Data on vital statuses and dates of death were obtained from the Korean national registry of vital statistics. Follow-up transthoracic echocardiographic evaluations were generally performed at six-month intervals in the first year and every 2 years thereafter. Early mortality was defined as death within 30 days of surgery. Deaths were classified as cardiac or non-cardiac based on medical records. The definition of valve-related events was based on the Guidelines for Reporting Mortality and Morbidity after Cardiac Valve Interventions [19].

### Echocardiography

Two-dimensional and Doppler echocardiographic examinations were performed using HP Sonos 5500 (Hewlett-Packard; Andover, USA), Philips iE33 (Philips Medical Systems, Bothell, USA), and GE vivid 7, E9 (GE Medical System, Horten, Norway). Preoperative echocardiography was performed in all patients less than 2 months prior to the surgery. MR was graded as either moderate (0.2 cm2 ≤ ERO < 0.4 cm2) or severe (ERO ≥ 0.4 cm2). MV areas were estimated using the pressure half-time method. Significant pulmonary hypertension was defined as a tricuspid regurgitation peak velocity > 3.4 ms − 1, equal to a pulmonary artery pressure > 50 mmHg. Late mitral dysfunction was defined as the occurrence of a deterioration in severity; in other words, (1) the progression from mild dysfunction to moderate dysfunction, (2) from mild dysfunction to severe dysfunction, or (3) from moderate dysfunction to severe dysfunction.

### Statistical analysis

Categorical variables are presented as frequencies and percentages, and continuous variables are expressed as means ± SD or as medians and ranges. Kaplan–Meier curves were employed to delineate overall survival, valve-related event-free survival, and freedom from mitral dysfunction. Stratified survival curves were plotted to determine unadjusted differences for variables of interest (log-rank test). For multivariate analyses, the Cox proportional hazards model was used to determine the association of baseline characteristics with time to MV dysfunction. Pre-specified covariates (Table 1) and the presence of postoperative atrial fibrillation were included in this analysis. Variables with a probability value of < 0.20 in univariate analyses were chosen as candidates for analyses with the multivariate Cox proportional hazards model. Results are expressed as hazard ratios (HR) with 95% confidence intervals (CI). *p* values < 0.05 were considered significant. For further verification of the results of Cox-regression analysis, the model was validated in 1000 bootstrap samples. SPSS version 18.0 (SPSS Inc., Chicago, IL, USA) was used for all statistical analyses.

**Table 1:** Baseline characteristics of patients

	No. of cases (%) or mean ± SD
Number of patients	292
Age (years)	61.9 ± 13.0
Female gender (n, %)	113 (38.7)
Body mass index	23.3 ± 3.5
NYHA class	
III	177 (60.6)
IV	34 (11.6)
Underlying condition (n, %)	
Hypertension	98 (33.6)
Diabetes mellitus	29 (9.9)
COPD	8 (2.7)
History of thromboembolic events	7 (2.4)
Creatinine, mg/dL	1.2 ± 1.3
Dialysis	11 (3.8)
Preoperative atrial fibrillation	41 (14.0)
LV ejection fraction (%)	51.4 ± 13.6
Significant pulmonary hypertension	60 (20.5)
Etiology of mitral valve	
Rheumatic	67 (22.9)
Non-Rheumatic	225 (77.1)
Functional	124 (42.5)
Degenerative	101 (34.6)
Mitral valve lesion (n, %)	
Predominant mitral stenosis	19 (6.5)
Predominant mitral regurgitation	259 (88.7)
Mixed steno-regurgitation	14 (4.8)
Aortic valve lesion (n, %)	
Predominant aortic stenosis	100 (34.2)
Predominant aortic regurgitation	118 (40.4)
Mixed steno-regurgitation	74 (25.3)

*NYHA* New York Heart Association, *EF* ejection fraction, *COPD* chronic obstructive pulmonary disease, *LV* left ventricle

## Results

### Baseline characteristics

The mean age of patients at surgery was 61.9 ± 13.0 years and 38.7% (*n* = 113) of the patients were females. Seventy-two percent (*n* = 211) of the patients were categorized under New York Heart Association functional class III or IV, and the etiology of MV was a rheumatic (*n* = 67, 22.9%) or non-rheumatic (*n* = 225, 77.1%) lesion (Table 1). A Maze operation was performed concomitantly in the patients with atrial fibrillation (*n* = 9).

### Early outcomes

There were three (1.0%) early deaths: two patients died of postoperative low cardiac output syndrome and one patient died of postoperative hypovolemic shock. There were nine cases of early postoperative complications, including postoperative bleeding in three (1.0%) patients, paravalvular leakage in two (0.7%) patients, seizure in one (0.3%) patient, and wound problems in three (1.0%) patients.

### Late outcomes

Clinical follow-up was 100% (*n* = 292) with a mean follow-up duration of 56.9 ± 46.5 months. There were 23 (7.9%) late deaths: 14 of these were cardiovascular-related and 9 were not cardiovascular-related. The causes of cardiovascular-related deaths were unknown in 12 patients and congestive heart failure in 2 patients. Non-cardiovascular causes of death were malignancy in 7 patients, sepsis in 1 patient, and intracranial hemorrhage in 1 patient. Overall survival at 5 and 10 years were 90.7% ± 2.1 and 87.2% ± 2.8%, respectively.

During the follow-up period, 28 patients experienced valve-related events, including 7 patients with valve-reoperation, 7 patients with anticoagulation-related bleeding, 5 patients with thromboembolisms, and 5 patients with infective endocarditis. The causes of reoperation included paravalvular leakage of previous prosthetic aortic valve in 3 patients, infective endocarditis in 1 patient, dysfunction of previous aortic bioprosthetic valve in 5 patients, severe tricuspid regurgitation in 2 patients, thoracoabdominal aortic aneurysm in 1 patient, aortic root dilatation in 1 patient, and severe MR in 1 patient. Freedom from valve-related events at 5 and 10 years were 94.8 ± 1.5% and 78.9 ± 5.2%, respectively. Of these 14 reoperations, 2 were MV-related reoperations. The causes of MV-related reoperation were native mitral infective endocarditis in 1 patient and severe MR in another. Freedom from MV-related reoperation at 5 and 10 years were 99.5 ± 0.5% and 98.6 ± 1.0%, respectively.

### Mitral valve dysfunction

**Fig. 1:**
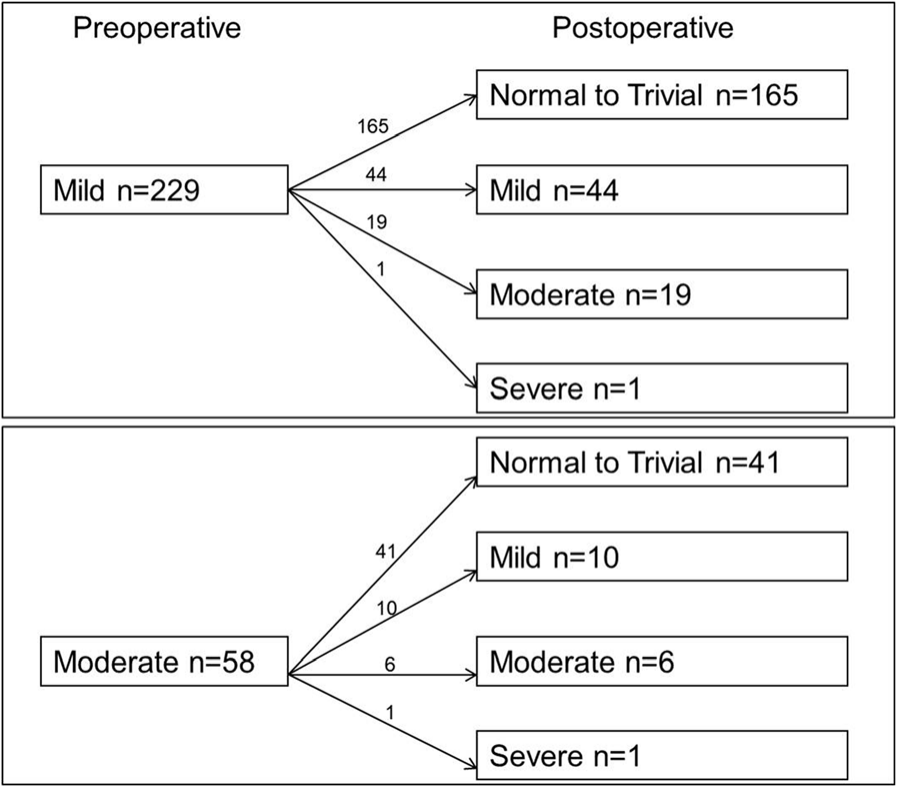
Postoperative changes in the severity of mitral valve dysfunction after isolated aortic valve replacement

**Fig. 2:**
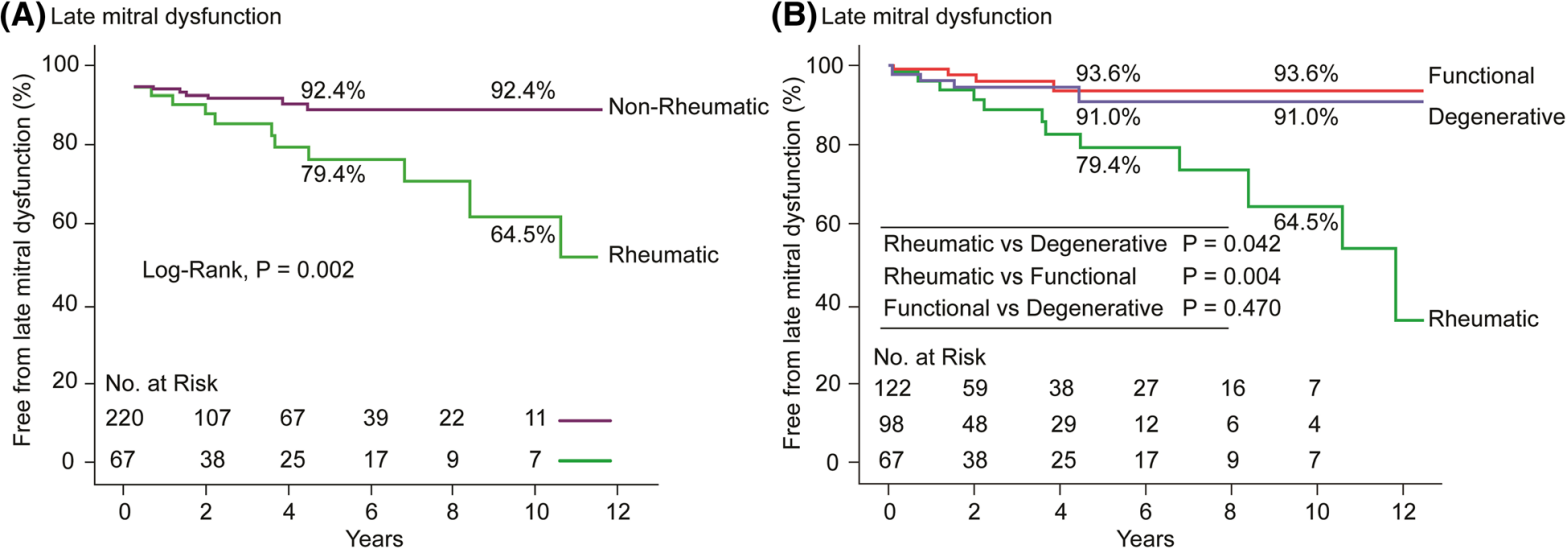
Kaplan-Meier curves for freedom from mitral dysfunctions based on the etiology of mitral valve disease: **a** non-rheumatic vs. rheumatic; (**b**) functional vs. degenerative vs. rheumatic

A total of 229 (78.4%) patients were evaluated with echocardiography for more than 6 months postoperatively. During a mean echocardiography follow-up duration of 40.8 ± 44.5 months, 21 patients experienced the progression of late mitral dysfunction. The changes in MV dysfunction after isolated AVR during the follow-up period are shown in Fig. 1. Freedom from late mitral dysfunction at 5 and 10 years were 88.8% ± 2.7 and 83.6% ± 4.5%, respectively. The etiology of MV disease (rheumatic vs. non-rheumatic) was evaluated using a Cox model, with the rheumatic origin emerging as a significant risk factor for MV dysfunction, even in univariate analysis (*p* = 0.006, Table 2, Fig. 2). Based on multivariate analysis, rheumatic pathology of MV (HR: 3.88, 95% CI 1.60–9.39, *p* = 0.003) was an independent predictor of late mitral dysfunction.

**Table 2:** Univariate and multivariate analyses of mitral valve dysfunctions

	Univariate	Multivariate	
	*p* value	Hazard ratio (95% CI)	*p* value
Preoperative factors			
Age	0.340		
Female gender	0.191		
NYHA functional class > 2	0.195	2.88 (0.83–10.05)	0.096
Hypertension	0.228		
Diabetes mellitus	0.119		
History of thromboembolic events	0.758		
Creatinine, mg/dL	0.393		
Dialysis	0.286		
Preoperative atrial fibrillation	0.945		
Rheumatic etiology of mitral valve vs. Non-Rheumatic	0.006	3.88 (1.60–9.39)	0.003
Aortic valve lesion	0.869		
LV ejection fraction, %	0.127		
LV end-systolic dimension, mm	0.066		
LV end-diastolic dimension, mm	0.174		
Significant pulmonary hypertension	0.930		
Operative factors			
Aortic valve type	0.653		
Tricuspid annuloplasty	0.624		
Maze procedure for AF	0.651		

*CI* confidence interval, *NYHA* New York Heart Association, *AF* atrial fibrillation, *LV* left ventricle

## Discussion

This study demonstrated that 75.3% of the patients with mild-to-moderate mitral dysfunction after isolated AVR improved, 17.4% remained unchanged, and 7.3% experienced worsening of mitral function over the long-term follow-up period. Multivariate analysis revealed that rheumatic etiology was a risk factor associated with increased mitral dysfunction, while degenerative mitral pathology showed a similar trend in functional mitral pathology, with a better postoperative mitral function compared with rheumatic pathology.

Several studies have mainly considered functional MR with respect to mitral function after isolated AVR [7, 20, 21]. In contrast, very few studies have included organic mitral disease [5, 6, 13, 17, 18]. Tunick et al. analyzed echocardiography data, including organic etiology, from 27 patients (≥mild MR) and reported that the severity of preoperative MR itself was associated with postoperative improvement in MR, without mentioning the correlation between mitral pathology and MR improvement [5]. Brasch et al. studied 27 patients undergoing isolated AVR, observing mitral annular calcification in 26 patients and leaflet thickening in 9 patients as mitral organic pathologies. They demonstrated that a larger preoperative left ventricle (LV) mass was the only significant predictor of improvement in MR. However, they did not find significance in mitral organic pathology [6]. In a postoperative echocardiographic review, Barreiro et al. studied 70 patients with at least moderate MR undergoing AVR. They found that mitral organic disease was associated with a poor improvement in MR compared with functional mitral disease. However, in that study, postoperative echocardiograms of only 37 patients were available for review [13]. Vanden Eynden et al. studied 80 patients with at least moderate MR, including 64 patients with an organic mitral pathology at 1 year post-AVR. They found that ischemic and functional MR improved after isolated AVR, whereas rheumatic and myxomatous disease remained stable or deteriorated. They concluded that the etiology of MR was a significant factor involved in improvement in MR [17]. Unger et al. reviewed quantitative echocardiographic data of 52 patients with at least mild MR after AVR. They found that on the eighth day post-AVR, the postoperative improvement in MR was mainly related to the severity of preoperative MR and the extent of mitral coaptation height, regardless of the etiology of MV [18]. These previous studies were limited by their small study populations and relatively short durations of echocardiography follow-up, ranging from 8 days to 1 year after the surgery. The present study reviewed 292 patients with mild-to-moderate mitral dysfunction (etiology of MV: functional in 124 patients, degenerative in 101, and rheumatic in 67), allowing for statistical analysis. Moreover, because organic mitral pathology is a structural and progressive lesion, as well as an organic lesion that could contribute to mitral dysfunction even after the correction of LV afterload through AVR, the long-term status of conservatively treated organic MVs remains questionable. Therefore, long-term echocardiographic evaluation is required to assess progressive mitral dysfunction over time post-AVR. The current study had a longer duration of echocardiographic data than other studies, with a mean echocardiography follow-up duration of 40.8 ± 44.5 months.

The present study revealed that rheumatic mitral disease was an independent factor associated with postoperative mitral dysfunction after isolated AVR. This finding is consistent with a study by Vanden Eynden et al. [17]. In the current study, 21% of the patients with rheumatic mitral disease experienced late mitral dysfunction at 5 years after AVR, whereas only 8% of those with non-rheumatic mitral lesion experienced late mitral dysfunction at the same time of follow-up (*p* = 0.002). Rheumatic MV disease is a progressive lesion and this etiology can affect mitral dysfunction over time after isolated AVR. Moreover, postoperative MR (≥moderate) affected outcomes of poor long-term survival [13]. Therefore, patients with mild-to-moderate MR of rheumatic origin should be considered for simultaneous MV surgery at the time of AVR.

## Limitations

This study was subjected to the limitations inherent in a retrospective study using observational data from a single center. Another limitation is that late (> 6 months) postoperative echocardiography data were not available for 21.6% of the patients.

## Conclusions

In conclusion, conservatively treated patients with mild-to-moderate mitral dysfunction exhibited acceptable clinical outcomes. Rheumatic pathology of MV is associated with a higher risk of progressive native MV dysfunction. Therefore, patients with mild-to-moderate MR of rheumatic origin should be considered for simultaneous MV surgery at the time of AVR.

## Data Availability

The datasets generated and analyzed during the current study are available from the corresponding author on reasonable request.

## Abbreviations

AF: Atrial fibrillation
AVR: Aortic valve replacement
CI: Confidence interval
COPD: Chronic obstructive pulmonary disease
EF: Ejection fraction
ERO: Effective regurgitant orifice
INR: International normalized ratio
LV: Left ventricle
MR: Mitral regurgitation
MV: Mitral valve
NYHA: New York Heart Association
